# Pectus excavatum in motion: dynamic evaluation using real-time MRI

**DOI:** 10.1007/s00330-022-09197-1

**Published:** 2022-10-29

**Authors:** Daniel Gräfe, Martin Lacher, Illya Martynov, Franz Wolfgang Hirsch, Dirk Voit, Jens Frahm, Harald Busse, Sergio Bruno Sesia, Sebastian Krämer, Peter Zimmermann

**Affiliations:** 1 Department of Pediatric Radiology, University Hospital, Leipzig, Germany; 2 Department of Pediatric Surgery, University Hospital, Leipzig, Germany; 3 Biomedizinische NMR, Max-Planck-Institut für Biophysikalische Chemie, Göttingen, Germany; 4 Department of Diagnostic and Interventional Radiology, University Hospital, Leipzig, Germany; 5 Division of General Thoracic Surgery, Bern University Hospital, Bern, Switzerland; 6 Division of General Thoracic Surgery, Department of Visceral, Transplant, Thoracic and Vascular Surgery, University Hospital, Leipzig, Germany

**Keywords:** Funnel chest, Magnetic resonance imaging, Thoracic wall

## Abstract

**Objectives:**

The breathing phase for the determination of thoracic indices in patients with pectus excavatum is not standardized. The aim of this study was to identify the best period for reliable assessments of morphologic indices by dynamic observations of the chest wall using real-time MRI.

**Methods:**

In this prospective study, patients with pectus excavatum underwent morphologic evaluation by real-time MRI at 3 T between January 2020 and June 2021. The Haller index (HI), correction index (CI), modified asymmetry index (AI), and modified eccentricity index (EI) were determined during free, quiet, and forced breathing respectively. Breathing-related differences in the thoracic indices were analyzed with the Wilcoxon signed-rank test. Motion of the anterior chest wall was analyzed as well.

**Results:**

A total of 56 patients (11 females and 45 males, median age 15.4 years, interquartile range 14.3–16.9) were included. In quiet expiration, the median HI in the cohort equaled 5.7 (4.5–7.2). The median absolute differences (Δ) in the thoracic indices between peak inspiration and peak expiration were ΔHI = 1.1 (0.7–1.6, *p* < .001), ΔCI = 4.8% (1.3–7.5%, *p* < .001), ΔAI = 3.0% (1.0–5.0%, *p* < .001), and ΔEI = 8.0% (3.0–14.0%, *p* < .05). The indices varied significantly during different inspiratory phases, but not during expiration (*p* > .05 each). Furthermore, the dynamic evaluation revealed three distinctive movement patterns of the funnel chest.

**Conclusions:**

Real-time MRI reveals patterns of chest wall motion and indicate that thoracic indices of pectus excavatum should be assessed in the end-expiratory phase of quiet expiration.

**Key Points:**

*• The thoracic indices in patients with pectus excavatum depend on the breathing phase.*

*• Quiet expiration represents the best breathing phase for determining thoracic indices.*

*• Real-time MRI can identify different chest wall motion patterns in pectus excavatum.*

**Supplementary Information:**

The online version contains supplementary material available at 10.1007/s00330-022-09197-1.

## Introduction

Pectus excavatum is the most common chest wall deformity, with incidence ranging between 1:400 and 1:1000. Males are 3–5 times more affected than females [1]. Besides an impaired body image and a decreased quality of life [2], funnel chest may result in reduced cardiac function [3] and lung capacity [4]. Correction is often sought by patients with more severe forms of funnel chest. Therefore, a description of the location, extent, and depth of the funnel as well as the symmetry of the thorax is essential for therapeutic planning. Compared to CT, chest MRI not only provides the possibility for functional studies but also offers the advantage of imaging without ionizing radiation. The latter is particularly important for children and adolescents [5].

The most widely applied thoracic index for assessing the severity of pectus excavatum with cross-sectional images is the Haller index (HI) or pectus severity index [6]. A more recent index, the correction index (CI), often better reflects the degree of a funnel chest [7] as it is independent of the width of the thorax. Different metrics exist to quantify thoracic asymmetry, with the asymmetry index (AI) [8] and the eccentricity index (EI) [9] being among the more commonly used.

Since standard CT and MRI examinations require breath-hold maneuvers, little is known about chest wall movement in funnel chest patients under free breathing conditions. Until now, static axial cross sections of the thorax at the deepest point of the funnel have been used to determine the degree of pathology. However, the phase of the breathing cycle used for calculating the indices is not standardized, though there is evidence that HI and CI change during breathing [10]. Variation in the extent of breathing is probable, especially in children and adolescents, even if a certain breathing phase is preselected.

Real-time gradient-echo MRI now allows for dynamic monitoring of thoracic movements during free breathing at a high temporal and spatial resolution [11]. The real-time imaging approach offers general advances in pediatric imaging, as any type of movement is largely frozen, be it macro movement of uncooperative children or chest motion in insufficient breathing compensation [12]. Moreover, in comparison with other ultrafast sequences, MRI techniques with short gradient-echo times are only minimally sensitive to magnetic field inhomogeneities and susceptibility differences, which is especially relevant for thoracic imaging.

The specific aims of our study are:

(i) To determine pectus excavatum indices during the entire breathing cycle
(ii) To assess thoracic indices within defined breathing phases
(iii) To identify typical patterns of chest wall motion that might influence the indices

## Materials and methods

### Patient population

**Fig. 1:**
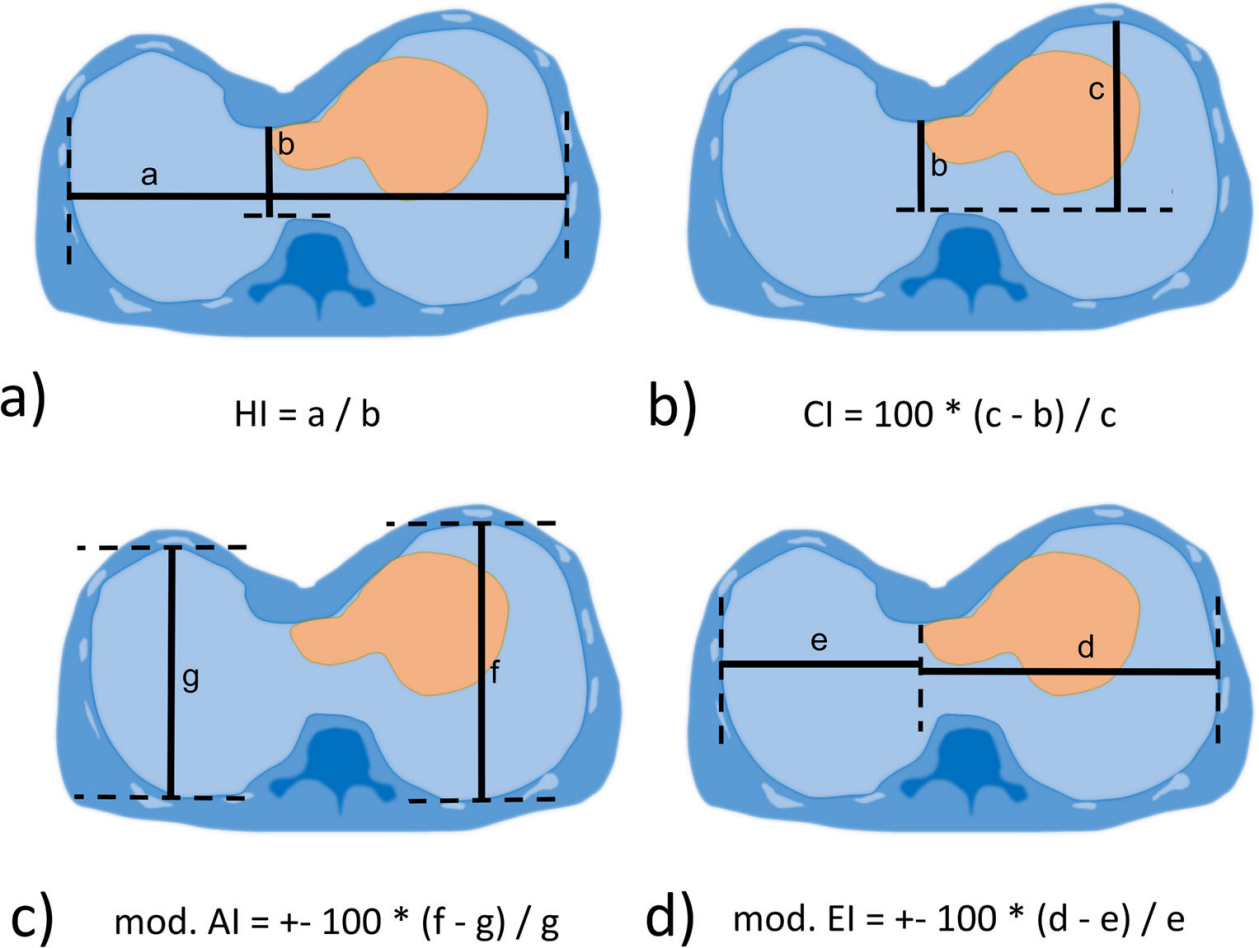
Schematic illustration of distances (solid lines) and definitions of (**a**) Haller index (HI), (**b**) correction index (CI), (**c**) modified asymmetry index (AI), and (**d**) modified eccentricity index (EI). Dashed lines are auxiliary contours

For this prospective study, all patients with pectus excavatum between 6 and 30 years of age, who presented for chest wall deformities in the Department of Pediatric Surgery between January 2020 and June 2021, were included. Real-time MRI of the thorax was performed to determine the thoracic dimensions and indices HI, CI, AI, and EI. Exclusion criteria included contraindications to MRI, such as claustrophobia and carrying metallic or electronic implants incompatible with MRI. Patients with a sterno-vertebral distance of less than 5 mm were excluded, assuming that the limited spatial resolution would have a considerable impact on index measurement (Fig. 1). The study was approved by the Institutional Review Board (169–20ek), and written consent was obtained from all subjects or their legal guardians.

### Real-time MRI

All examinations used 3 T (Prisma Fit, Siemens Healthcare) utilizing an 18-channel thorax coil in combination with suitable elements of an integrated 32-channel spine coil. Details of the real-time MRI technique have been described elsewhere [13]. In short, accelerated imaging is achieved by highly under-sampled spoiled gradient-echo sequences with radial k-space sampling and serial image reconstruction by nonlinear inversion with temporal regularization. Online reconstruction and image display are ensured by an integrated GPU computer, automatically bypassing the commercial reconstruction pipeline without manual interference.

After the localizer sequences for spatial orientation and identification of the deepest point of deformity, the protocol employed a dynamic real-time fast low-angle shot (FLASH) MRI sequence which simultaneously provided a sagittal and transverse image series in a frame-interleaved manner. After previous coaching of the patient (Supplemental Table 1), the measurement involved two sequential scans to monitor both quiet and forced breathing during periods of 13 s (corresponding to 2 × 200 frames). The experimental parameters included repetition time, 2.22 ms; echo time, 1.44 ms; 15 radial spokes; flip angle, 8°; slice thickness, 6 mm; field of view, 320 × 320 mm^2^; in-plane resolution, 1.6 × 1.6 mm^2^; and acquisition time per frame, 33.3 ms. The total examination time for the study sequences was 3 min, including scout sequences and teaching of breathing commands (MRI exam protocols are available as Supplemental Files). As a typical example, Supplemental Video 1 shows simultaneously acquired sagittal and transverse MRI movies of a patient with funnel chest during forced breathing.

### Thoracic indices

For quiet and forced breathing conditions, the dimensions required for calculating the thoracic indices HI, CI, AI, and EI were determined from transverse images at the deepest point of the funnel (Fig. 1). This task was performed by a pediatric radiologist (D.G., 12 years of experience in pediatric MRI) with use of a conventional DICOM Viewer (syngo.plaza, Siemens). For assessment of interobserver variability, the analysis was independently performed by a study nurse (I.K., 8 h of training). Values of HI > 3.25 and CI > 28% were presumed as pathological according to the literature [14]. A correlation analysis between HI and CI was performed. Modified AI and EI measures (Fig. 1) were introduced to account for different lateralities, with 1 being subtracted from the ratio of larger diameter and smaller diameter. Right laterality was tagged with a negative sign.

### Morphologic classification

Participants were grouped using the morphologic classification of the pectus excavatum proposed by Park et al [15] which distinguishes between symmetric and asymmetric funnels (Supplemental Fig. 1). We simplified the existing scheme to differentiate by symmetry only, i.e., Park 1 or Park 2. Classification of the participants was performed using transverse images by two senior pediatric surgeons (P.Z. and S.B.S., both with over 10 years of experience in surgery of pectus excavatum) and a pediatric radiologist (D.G.) in independent sessions.

### Movement patterns

For further examination of thoracic wall movements, the deepest point of the funnel and the highest points of the thorax on both sides were continuously tracked during forced beathing, using an open-source software (Tracker version 5.1.5, https://physlets.org/tracker/). Curves were smoothed with a cubic spline algorithm for visualization purposes (in R Studio version 1.2.5033 [RStudio Team 2020], RStudio: Integrated Development for R. RStudio, PBC).

### Statistics

The interobserver variability of the thoracic dimensions was assessed with intraclass correlation coefficients. The distribution of values for different breathing phases was tested for normality using the Shapiro-Wilk test. Due to the absence of normal distributions in our groups, differences in the central tendency for quiet and forced inspiration or expiration were assessed by a Wilcoxon signed-rank test. Numeric results are given as median and interquartile range. For associations of the movement patterns with Park’s morphological classification, Fisher’s exact test was applied. The interobserver variability of the Park classification was analyzed with Fleiss’ kappa. Agreement was rated as sufficient (kappa values 0.21–0.40), moderate (0.41–0.60), substantial (0.61–0.80), and excellent (0.81–1.00), as suggested by Landis and Koch [16]. Correlation of the indices as well as the change of indices during breathing cycle was evaluated with Pearson’s correlation coefficient. A correction for multiple comparisons applied with Holm-Bonferroni. Statistical analyses were conducted with RStudio (details given above) assuming a significance level of 0.05.

## Results

### Study population

A total of 56 subjects (11 females and 45 males, median age 15.4 years, interquartile range 14.3–16.9) were included. One patient with a sterno-vertebral distance of below 5 mm was excluded. The population could be classified as follows: out of the 56 participants, 55% (31) had a symmetric funnel chest (Park 1A and 1B) and 45% (25) an asymmetric one (Park 2A–C). Agreement between the three readers for morphologic classification was moderate (*κ* = 0.42), but substantial when limiting grouping into symmetrical or asymmetrical forms (*κ* = 0.61). In quiet expiration, the median values were HI = 5.7 (4.5–7.2), CI = 37% (30–43%), AI = − 2% (− 8 to 2%), and EI = 12% (− 6 to 35%).

### Index variation during breathing

**Fig. 2:**
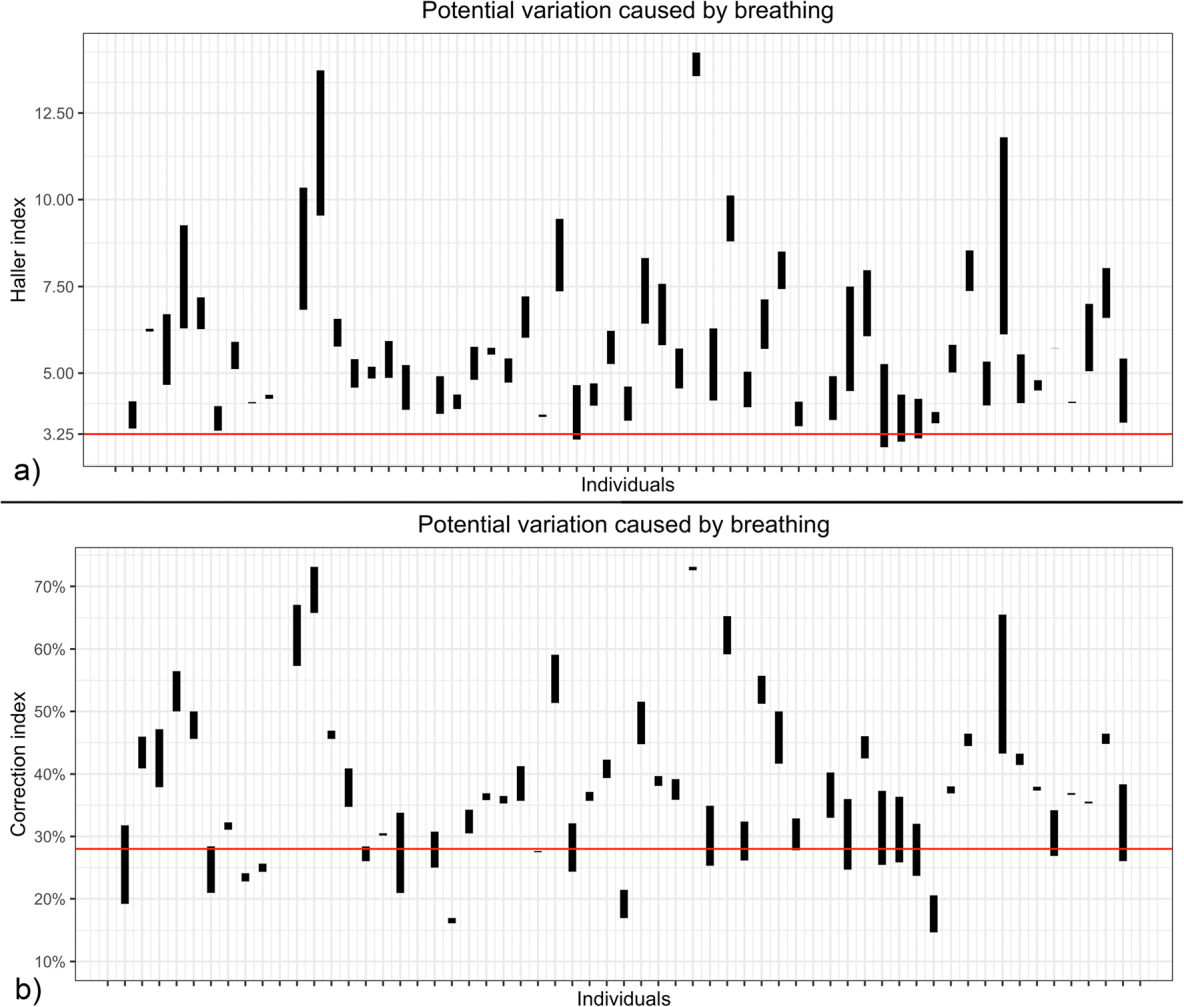
Ranges of HI and CI during maximum inspiration and expiration for all 56 participants. Four (7%) subjects crossed an HI of 3.25, which is considered a pathologic threshold. Fifteen (27%) subjects crossed the corresponding CI value of 28%

**Fig. 3:**
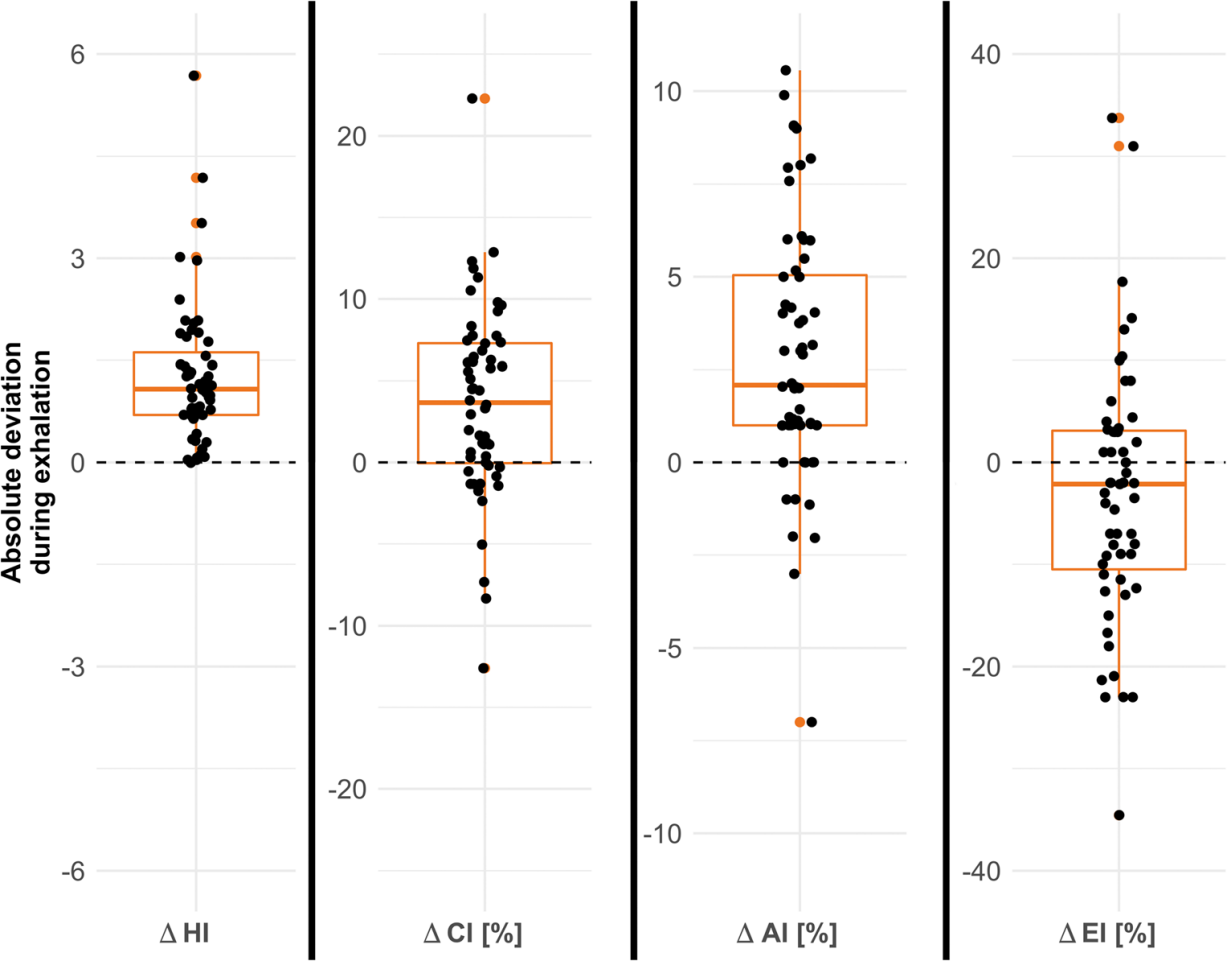
Absolute deviations between peak inspiration and expiration for HI, CI, AI, and EI. While HI increased in 98% of participants during expiration, CI decreased in 25%. AI tended to increase with expiration. The EI did not show a directional change

**Fig. 4:**
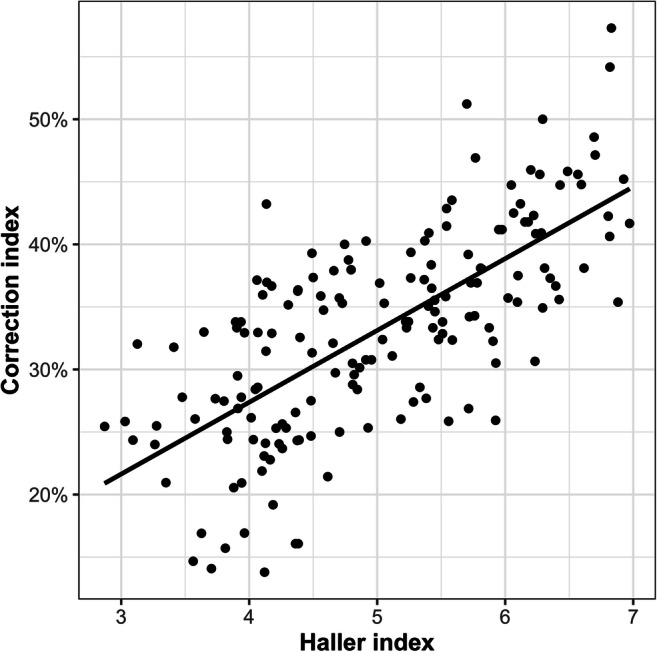
Correlation between HI and CI. Although there is a linear relationship, the dispersion is rather large (*r*^2^ = 0.48, *p* < .001)

All thoracic indices showed significant differences (Δ) between maximal inspiration and maximal expiration (Fig. 2). During exhalation, HI was found to be higher in 55 subjects (98%), while CI was lower for 14 (25%). The impact of breathing resulted in 4 subjects (7%) having HI values above the pathologic threshold of 3.25 and 15 subjects (27%) passing the CI threshold of 28%. Figure 3 shows the median absolute deviation of the thoracic indices during forced expiration, which were ΔHI = 1.1 (0.7–1.6, *p* < .001), ΔCI = 4.8% (1.3–7.5%, *p* < .001), ΔAI = 3.0% (1.0–5.0%, *p* < .001), and ΔEI = 8.0% (3.0–14.0%, *p* < .05). The correlation between HI and CI was weak (*r*^2^ = 0.49, *p* < .001) (Fig. 4). The interrater variability for the assessment of the morphologic indices was excellent, with intraclass correlation coefficients being 0.98 for HI, 0.97 for CI, 0.95 for AI, and 0.94 for EI. There was no significant correlation between HI and ΔCI, ΔAI, or ΔEI. Neither was found a correlation between age of the patient and the chest indices.

### Residual variation of breathing phases

For inspiration, significant differences were found in the measured indices depending on the extent of inspiration. The median values for the deviation were HI = 0.9 (0.4–1.4, *p* < .001), CI = 4.7% (2.6–6.9%, *p* = .001), AI = 2.2% (1.0–4.2%, *p* < .05), and EI = 7.5% (3.0–13.2%, *p* < .05). In contrast, during expiration, the differences according to depth of expiration were not significant and yielded HI = 0.3 (0.1–0.5, *p* = .19), CI = 2.9% (0.7–4.0%, *p* = .14), AI = 2.2% (1.1–4.0%, *p* = .48), and EI = 6.0% (2.0–12.6%, *p* = .50). No significant correlation was found between age of the patient and ΔHI, ΔCI, ΔAI, or ΔEI during the breathing cycle.

### Movement patterns

**Fig. 5:**
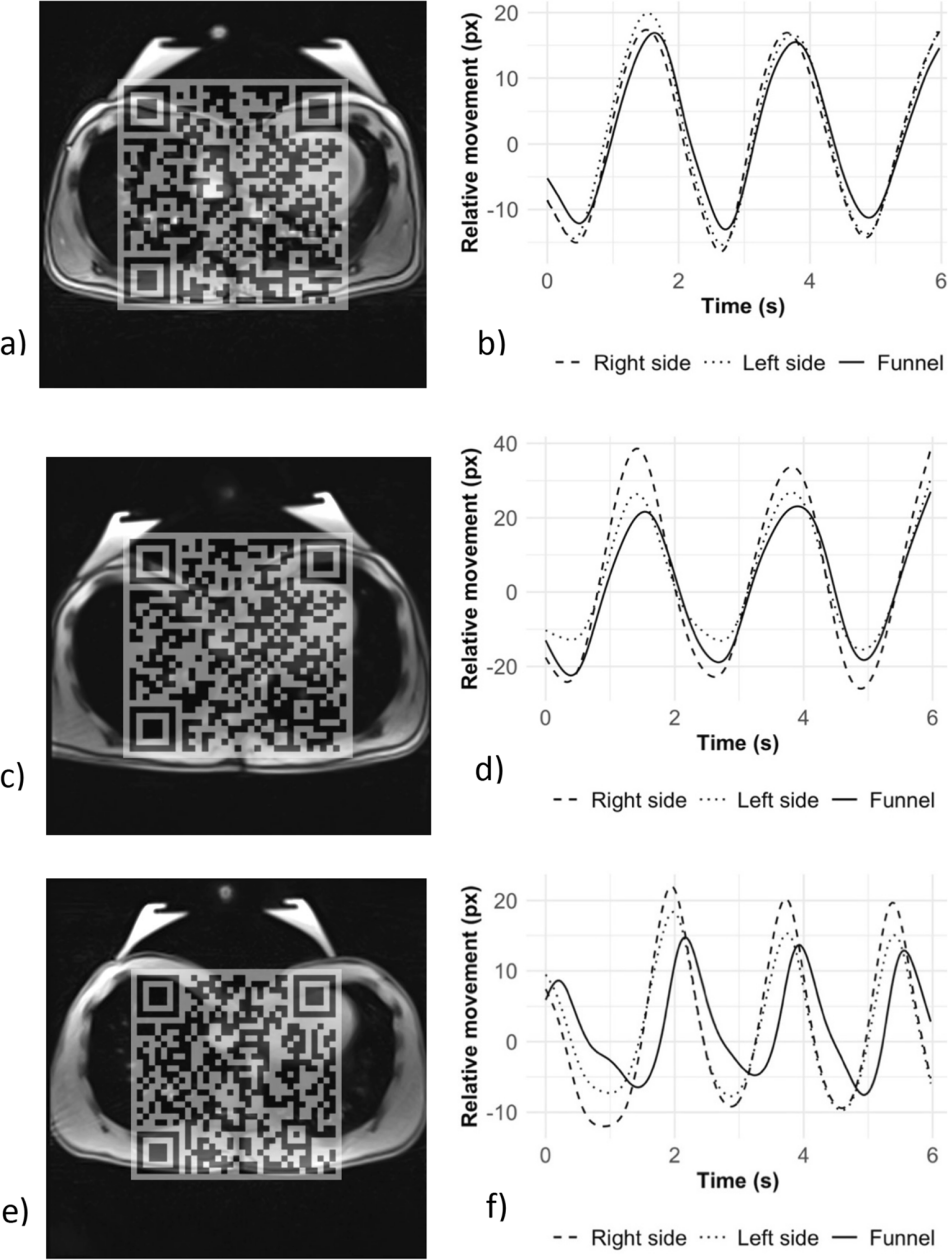
Three distinct movement patterns characterized by selected frames of MRI videos and corresponding tracking curves in our cohort of patients with funnel chest. Please take note of the deactivated vacuum bell placed upon the funnel as part of our standard estimate of treatment response using real-time MRI. (Video 5a, Fig. 5b) Symmetric movement of both sides and funnel (Video 5c, Fig. 5d) pronounced movement of the lower side in asymmetric pectus excavatum, and (Video 5e, Fig. 5f) delayed movement of one side of the funnel

Sufficient motion data was acquired for 49 participants. Three patients were excluded because MRI videos were only available for one breathing cycle and a reliable determination of movement patterns was not possible. Four patients were excluded due to irregular breathing. Three predominant movement patterns were identified (Fig. 5). The most prevalent variant in 25 of 49 subjects (51%) was a largely uniform movement of both sides of the thorax and the funnel. This pattern was found in patients with a symmetrically as well as asymmetrically shaped funnel chest (14 of 25, 56%, in Park 1 and 11 of 25, 44%, in Park 2). The second most common movement pattern in 15 of 49 participants (31%) exhibited increased motion of only one side of the thorax (11 of 15 on the right, 4 of 15 on the left). The greater movement was frequently seen on the side of an asymmetrically located funnel. As a third pattern, a delayed motion of the funnel compared to both sides was found in 9 of 49 participants (18%) both in symmetric and asymmetric funnel chests (4 of 9, 44% in Park 1 and 5 of 9, 46% in Park 2). For associations of the movement patterns with the morphological Park classification, the level of significance was not reached.

## Discussion

The human thorax is one of the most dynamic regions of the body. Static imaging only delivers temporal snapshots with limited information. Real-time MRI puts a dynamic evaluation of the thorax morphology during free breathing into practice, which is of particular interest in patients with chest wall deformities. Moreover, free breathing may improve the reproducibility of different thoracic index measurements. During quiet breathing, the resting position at the end of quiet expiration is more reproducible than under command-driven forced breathing. This holds especially true in children who tend to exaggerate breath-hold commands. Furthermore, very deep inspiration and expiration are tracked more accurately in free breathing as the dynamic retraction forces of the thorax hinder measurements during breath holding. It should also be mentioned that, apart from their significance for therapeutic decision-making, thoracic indices also play an important role in reimbursement in some health care systems. Therefore, it is essential to be aware of index variations during different breathing conditions.

The present work demonstrates that breathing significantly influences all four standard indices. Seven participants crossed the threshold of 3.25 for HI and 15 the threshold of 28% for CI, each considered a cut-off for surgical therapy by some surgeons [17, 18]. However, the significance of these results is limited since the number of patients crossing the threshold depends on whether the median of the patient cohort is near that threshold or not. In our cohort, on an individual basis, crossing those thresholds did not alter therapeutic management. Moreover, it is questionable whether the reported correlation between HI and CI [18], with an HI of 3.25 corresponding to a CI of 28%, is truly robust and depends on the chest width of the cohort selected. The correlation analysis of HI and CI in our study population showed an only moderate linear relation. Once a breathing phase for breath-hold measurements has been selected, deviations may still occur depending on how the participants follow the respective commands. We found significant variations for inspiration but not for expiration breath-hold.

But, in accordance with other studies [19, 20], we showed significant changes of HI and CI with breathing which highlights the need of standardization in the assessment of the thoracic indices of pectus excavatum. Especially in patients with “borderline” severity, the measurement in the end-expiratory phase of quiet expiration represents the real degree of the chest wall deformity and may lead to an increase in surgical candidacy.

Our results are in line with recent studies: Lollert et al examined 69 subjects with funnel chest with respect to HI, CI, and AI during both inspiration and expiration, using static fast spin echo images [10]. There was a clear correlation between breathing changes of HI and CI as both indices increased during expiration, in contrast to AI. The targeted depth of inspiration and expiration was not mentioned. Birkemeier et al evaluated HI and AI in 47 participants with funnel chest using steady-state free-precession images [20]. As in our study, the authors recorded a significantly lower HI in forced inspiration compared to both quiet breath-hold and forced expiration. AI was independent of the breathing phase and CI was not studied.

The agreement of the three readers over the Park classification of pectus excavatum was quite low but improved upon simplifying the scheme into symmetric and asymmetric funnel chests—the two clinically most relevant groups of pectus excavatum. One explanation for low concordance between the readers could be that the different shapes of pectus excavatum represent a broad spectrum, with wide transitions between centric and eccentric funnels as well as between narrow and broad funnels.

The examination of chest wall dynamics requires a motion analysis based on dynamic MRI. Using real-time MRI at a high spatiotemporal resolution, we identified three frequent movement patterns in our cohort. In 2006, Herrmann et al postulated three different movement patterns of the thoracic wall in funnel chest patients [21]. They used MRI sequences at 1 frame per second in forced inspiration and expiration breath-holds on 7 participants. However, the described patterns were not found in our larger cohort except for the “wing-beat” pattern, which matches a pattern observed in our study with delayed motion of the funnel. Since no apparent correlation of the movement patterns with the anatomical classification was observed, these patterns might represent independent prognostic markers—a task for future studies to address.

The use of dynamic real-time MRI in the assessment of chest wall movements and classification of pectus excavatum in pediatric patients was highly appreciated by the participating surgeons and helped in their decision-making. The clear visualization of the sternal depression on the heart and lungs are of high value for the achievement of the full diagnosis of the defect. Furthermore, in the future, the real-time changes under evaluation of the sternum by a vacuum bell could simulate nearly the conditions after repair are of high. Further, it enables the patient and the family to understand that the pectus excavatum is not only an esthetic problem and facilitates to deal with health insurance companies.

A limitation of this study is that the clinical impact of the measurement variance is not addressed. Furthermore, it cannot be ensured whether the participants carried out forced breathing commands with sufficient commitment, despite prior training of breathing maneuvers. However, this would result in an underestimation of the breathing variance.

We recommend measuring the thoracic indices of pectus excavatum during free quiet breathing using real-time MRI. If real-time MRI is not available, index assessments by conventional rapid scan MRI techniques should be performed in the end-expiratory phase of quiet expiration. These recommendations are based on the following observations: (a) during this phase, indices do not significantly depend on the degree of expiration, (b) respective indices exhibit a high reproducibility, and (c) they correspond most closely to the intraoperative situation. Further studies are required to elucidate any prognostic implication of the chest wall motion patterns described here.

## Supplementary information

ESM 1 (DOCX 295 kb)

ESM 2 (MP4 15072 kb)

## Abbreviations

AI: Asymmetry index
CI: Correction index
EI: Eccentricity index
HI: Haller index

## Declarations

### Guarantor

The scientific guarantor of this publication is Daniel Gräfe.

### Statistics and biometry

One of the authors has significant statistical expertise.

### Methodology

• prospective

• cross-sectional study

• performed at one institution
