# Supplementary material for: Pectus excavatum in motion: dynamic evaluation using real-time MRI
Source: Eur Radiol. 2022 Oct 29;33(3):2128–35. doi: 10.1007/s00330-022-09197-1 (PMC9935721; doi:10.1007/s00330-022-09197-1)
Supplement: Supplementary file 1 — (DOCX 295 kb) [file 330_2022_9197_MOESM1_ESM.docx]

**Electronic Supplementary Material**

**Supplemental Figure 1** Real-time MRI video of a patient with funnel chest during forced breathing. The (left) sagittal and (right) transverse image series are acquired simultaneously in a frame-interleaved manner with acquisition time per frame being 33 ms. The position of each plane is visible by a corresponding dark line of pre-saturated spins. Please take note of the non-activated vacuum bell placed upon the funnel as part of our usual estimate of treatment response using real-time MRI.

**Supplemental Fig. 2** Modified Park classification for pectus excavatum [15]. Park 1 for symmetric forms with subgroup 1A for narrow and 1B for broad funnels. Park 2 for asymmetric shapes with the following subgroups for lateralized funnels: 2A1 for narrow funnel, 2A2 for broad funnel, 2A3 for Grand Canyon type, 2B for central funnels but distinctly different thoracic heights, and 2C for a combination of 2A and 2B.


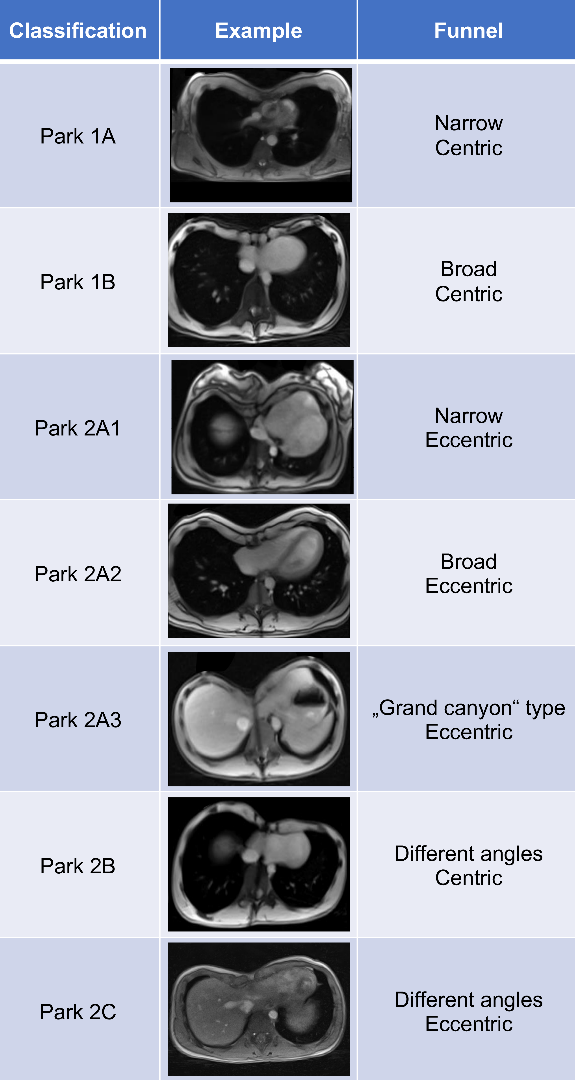


**Supplemental Table 1** Voice commands used for the study

| **Normal breathing** | No command |
| --- | --- |
| **Forced, deep breathing** | “Please breath in and breath out repeatedly as deep as you can, as long as the scanner is noisy” |

**Supplemental Files** MRI examination parameters as PDF file and exar1 file (Siemens binary) for the study protocol
